# Partial Replacement of Soybean Meal with Black Soldier Fly (*Hermetia illucens*) Larva Meal Maintains Stable Reproductive Performance and Health Status of Sows and Their Offsprings

**DOI:** 10.3390/vetsci13010002

**Published:** 2025-12-19

**Authors:** Vetriselvi Sampath, Kyejin Lee, In Ho Kim

**Affiliations:** 1 Department of Animal Biotechnology, Dankook University, Cheonan 31116, Republic of Korea; vetri09@dankook.ac.kr (V.S.); jookyjin1234@dankook.ac.kr (K.L.); 2 Smart Animal Bio Institute, Cheonan 31116, Republic of Korea

**Keywords:** black soldier fly, reproduction performance, milk composition, litter size, weaning piglets, insect meal

## Abstract

The objective of this study was to evaluate the responses of the reproductive performance, milk composition, and blood profile of sows, as well as the performance of their offspring when their dietary soybean meal is partially replaced by black soldier fly *Hermetia illucens* larva meal (BSFLM). A total of 18 multiparous (Landrace × Yorkshire) Duroc mated sows were randomly allocated to one of three dietary treatments based on parity from gestation to lactation on day 21. Each treatment has six replications. The dietary treatments were as follows: CON, basal diet based on corn-soybean meal; BSFLM1, basal diet soybean meal partially replaced with BSFLM (0.5% of diet); BSFLM2, basal diet soybean meal partially replaced with BSFLM (1.0% of diet). Our results revealed that sows fed with 0.5% of BSFLM had significantly increased (*p* < 0.05) BW at the initial and weaning period. Also, the BSFLM2 group sows showed significantly increased (*p* < 0.05) backfat thickness at weaning. Compared to CON, piglets born from the BSFLM group sows showed a tendency (*p* > 0.1) of having increased BW (at initial and at weaning) and ADG. Furthermore, sows fed with 0.5% BSFLM showed a tendency (*p* > 0.1) of having increased protein and fat % in milk at week 1. The serum concentration of IGF-1 was significantly *(p* < 0.05) increased in the BSFLM1 group sows compared to the BSFLM 2 group sows initially and it was equalized at weaning. In summary, partial replacement of soybean meals with 0.5% and 1% BSFLM could be beneficial for improving the reproductive traits, milk composition, and health status of sows and their offsprings.

## Simple Summary

As the global population continues to grow, the demand for meat, milk, and eggs is increasing. This puts pressure on livestock feed resources, especially protein sources like soybean meals (SBMs). SBM may become less available in the future and is also linked to environmental issues such as greenhouse gas emissions, deforestation, and soil degradation. To address these concerns, researchers are exploring more sustainable alternatives. One promising option is black soldier fly larvae (BSFL), which can efficiently convert organic waste into nutrient-rich protein while leaving a much smaller environmental footprint. Previous research in pigs has shown that a BSFL meal (BSFLM) can replace traditional protein ingredients without reducing growth and may even help support the immune system. However, very little is known about it. For this reason, our study investigated whether partially replacing SBM with small amounts of BSFLM (0.5% and 1.0%) in sow diets could influence the reproductive performance, blood health, milk quality, and growth of piglets.

## 1. Introduction

The rapid growth of the global population has a profound impact on both food security and environmental sustainability [1]. Rising demand for animal-derived foods such as poultry and pork is expected to intensify pig production, placing increased pressure on feed resources [2]. Soybean meal, the primary protein source in swine diets, is projected to face global shortages with this trend [3]. Beyond availability concerns, soybean cultivation is associated with notable environmental burdens, including greenhouse gas emissions, deforestation, and soil degradation [4]. These challenges highlight the urgent need to identify sustainable alternative protein sources that are suitable for large-scale swine production.

In this context, insects have increasingly gained attention as promising feed ingredients [5,6]. Among them, black soldier fly (*Hermetia illucens*) larvae (BSFL) stand out due to their low environmental footprint and ability to thrive on diverse organic waste substrates [7,8]. BSFL can convert organic waste into biomass within just one week, releasing only 28% of CO_2_ emissions compared to bacterial decomposition, which requires 45 days and emits about 50% CO_2_ [9]. They are not vectors of disease and offer an excellent nutritional profile, containing 37–63% protein with a favorable amino acid balance and high fat content [10]. Previous studies have demonstrated encouraging results: in weaned pigs, dietary inclusion of BSFL meal (BSFLM) at levels of up to 21% did not impair growth performance [11], while finishing pigs maintained normal performance with up to 8% replacement [12]. BSFLM supplementation has also been reported to enhance immune responses in weaned pigs [13].

Despite these promising findings, several limitations warrant consideration. A key challenge is the substantial variability in nutrient composition of BSFL, which is strongly influenced by rearing substrates, processing methods, and larval age [14]. Such variability can affect crude protein levels, amino acid balance, and lipid content, potentially leading to inconsistent performance outcomes when incorporated into swine diets. Moreover, differences in chitin content, a component known to influence digestibility, may further complicate diet formulation. These factors highlight the need for standardized production protocols and more comprehensive evaluations across different physiological stages. Notably, only one study [15] has examined the impact of the partial replacement of conventional protein sources (soybean meal, dried distillers’ grains with solubles, and fish meal) with BSFLM at 2.3% and 4.6% in sows. However, evidence regarding the partial replacement of BSFLM at lower levels in sows remains scarce, underscoring the importance of further research to determine its safety, efficacy, and optimal inclusion levels. We hypothesized that partial replacement of soybean meals with 0.5% and 1.0% BSFLM from gestation to lactation on day 21 would not compromise reproductive performance, blood parameters, or milk composition in sows, nor the growth performance of their piglets. Accordingly, the objective of the present study was to evaluate the impact of BSFLM supplementation on sow reproductive outcomes, blood profiles, and milk composition, as well as on piglet growth performance.

## 2. Materials and Methods

All the experimental procedures of the current study were approved by the Institutional Animal Care and Use Committee of Dankook University, Cheonan, Republic of Korea (DK-2-2326).

### 2.1. Animals, Husbandry, and Experimental Diets

This trial was carried out at Dankook University ‘Experimental Farm’ (Gonju-si, Korea). The standard farm management practices were adhered to for the sows and piglets. Estrus synchronization and artificial insemination (AI) were performed by direct exposure to boar twice a day (at 0800 and 1600 h). Estrus in sow was assumed when they showed a standing reaction in response to a back-pressure test while standing next to the boar. The study involved 18 sows (Landrace × Yorkshire) which were housed individually in a gestation crate measuring 2.02 × 0.70 m with autoloading feeding systems and they had ad libitum access to water. From 107th day of gestation, the sows were assigned to one of three dietary treatment groups in a completely randomized design based on their parity (average parity 3.2). Each treatment has six replicates of one sow and its litter per pen. The dietary treatments were as follows: CON, basal diet based on corn soybean meal; BSFLM1, basal diet soybean meal partially replaced with BSFLM (0.5% of diet); BSFLM2, basal diet soybean meal partially replaced with BSFLM (1.0% of diet). Sows were offered 4 kg of feed/day/sow before farrowing and 10 kg of feed/day/sow after farrowing. To calculate the average daily feed intake (ADFI) during gestation and lactation, the feed offered and remaining amount were recorded daily. This feeding strategy was applied from gestation to lactation on day 21. Daily rations for sows were divided into two parts: one part was given in the morning, and the remaining half was given eight hours later. BSFLM composition is provided in Table 1, and it was commercially obtained from Dodram Co., Ltd. (Seoul, Republic of Korea). Basal diets (Table 2) were formulated according to the recommendations of the NRC [16] for gestation and lactating sows. On day 114, sows were moved to farrowing crates (2.10 m × 1.8 m) and stayed until weaning on day 21. Feeding was postponed on the day of parturition. In the farrowing shed, a minimum 20 °C temperature was maintained until the end of the experiment. Heat lamps were used to provide the required additional heat for the piglets. After weaning, sows were fed 3.6 kg of feed daily until ovulation. Every newborn piglet received a 1 mL iron injection, within 24 h of birth. At the age of five, male piglets were castrated, and all piglets were ear-tagged. Piglets and sows both had unlimited access to water throughout the experiment, but during lactation, piglets were exclusively dependent on sow milk, and no creep feed was offered.

### 2.2. Sampling and Analysis

The metabolized energy (ME) of diets was measured by oxygen bomb calorimeter (Parr 6200, Parr Instrument Company, Moline, IL, USA). For crude protein analysis approximately 1 gm sample was taken into the glass tube with 12 mL concentrated H_2_SO_4_ and two Kjeltabs (3.5 g K_2_SO_4_ + 3.5 mg Se) tablets and digested at 420 °C for 45 min using Foss digester (Foss analytical, Hillerød, Denmark). Further distillation and titration were performed using the Kjeltec 2300 Nitrogen Analyzer (Foss Analytical, Hillerød, Denmark) following the product manual. It was multiplied by 6.25 to determine crude protein %. The calcium and phosphorus content of diets was analyzed by a spectrophotometer (Pharmacia, Cambridge, UK) after washing at 600 °C. Individual lysine and threonine composition of feed samples was measured by an amino acid analyzer (Sykam GmbH, Fürstenfeldbruck, Germany) after acid hydrolysis for 24 h in 6 N-HCl at 110 °C and evaporated at 50 °C in an evaporator (HAHNSHIN S&T Co., Ltd., Gyeonggi-do, Republic of Korea). Methionine was analyzed after overnight cold performic acid oxidation and subsequent hydrolysis.

Piglet birth weight, the total number of piglets born, and the number of piglets born alive were recorded for each litter. Also mummified fetuses and stillbirths were documented and added to the overall count of piglets born. The piglet birth weights were obtained by individually weighing them immediately after birth. Piglets were cross-fostered within groups to obtain the experimental litter size. The number of piglets per sow in all groups was set at 11–13 to ensure every litter had access to viable teats. The pre-weaning mortality percentage was determined by dividing the total number of piglets in each group by the number of piglets that died after cross-fostering.

The body weight of the sows was measured at beginning and at the end of weaning. Backfat thickness (BFT) on standing sows were measured after hair removal. The BFT was measured using the Minitube Backfat meter (AG0307SP) (Gyeonggi-do, Republic of Korea). The measurements were taken at the location of the last rib on each side of the sow, specifically at the P_2_ point, which is 65 mm from the midline. At weaning, the BFT was measured at the same marked spot on both sides of the sow. The assessment of backfat thickness involved calculating the difference between the backfat thickness measured at 107 days of gestation and the measurements taken at the point of weaning. Also, individual body weight of the piglet was recorded on the first day of birth and after 21 days of lactation (weaning) to determine their ADG.

One hour prior to milk sampling, piglets were separated from their dam. Then, the milk was collected by manual expression after administering a small dose of oxytocin (1–2 mL, intramuscular). Approximately 30 mL of milk was obtained from multiple functional teats of each sow, transferred to the laboratory, and subsequently stored at −20 °C. To examine proximate composition, including fat, protein, lactose, solids not fat (SNF), total solid, and freezing point, they were determined using a Milkoscan™ Mars analyzer (FOSS, Hillerød, Denmark).

Blood samples (10 mL/sow) were collected from each sow in non-anticoagulant tubes at the start and at weaning during the experiment. The samples were immediately centrifuged at 3000× *g* for 10 min to separate serum. Serum samples were then stored frozen (−20 °C) in glass vials until they were assayed. Serum cortisol concentration was analyzed using a commercially available Cortisol ELISA Kit (Boster Biological Technology, Pleasanton, CA, USA, Catalog: EK7119). Serum insulin-like growth factor-1 (IGF-1) was determined using a commercially available kit (Diagnostic System Laboratories Inc. Kits, Webster, TX, USA Catalog: DSL-5600). For Immunoglobulin G (IgG), samples were analyzed using a pig IgG ELISA kit according to the manufacturer’s instructions (ELISA Starter Accessory Package, Pig IgG ELISA Quantitation Kit; Bethyl, Montgomery, TX, USA). The serum Ca and P concentration was measured using the automated chemistry analyzer using a commercial kit by direct colorimetry (ELITech, Vitória, Espírito Santo—Brazil; Record 80171840050).

### 2.3. Statistical Analysis

The collected data for sow performance was analyzed using a general linear model of SAS^®^ version 9.4 (SAS Institute Inc., Cary, NC, USA). Dietary treatment was considered a fixed effect, whereas parity was included as a random effect. Tukey’s multiple range test was used for post hoc comparisons during treatment. To determine the effect of BSFLM administration, individual sows and their progenies (until d 21) were served as an experimental unit. For blood profile and milk composition data, individual sows were used as an experimental unit. A probability value < 0.05 was considered as significant, while 0.05 ≤ 0.10 was considered as tendency.

## 3. Results

The effects of dietary BSFLM on the reproduction performance of sows are shown in Table 3. Litter size, number of alive or dead piglets, stillbirth, mummified piglets, survival rate, and underweight piglet numbers did not differ between groups. However, BSFLM 2 group sows showed significantly (*p* > 0.05) increased BW initially and at the end of the weaning period. Furthermore, BFT improved significantly (*p* > 0.05) in BSFLM 2 group sows than the BSFLM1 group. The ADFI of both groups’ sows during gestation and lactation remained similar. Compared to CON, piglets born from the BSFLM group sows showed a tendency (*p* > 0.1) to have increased BW (initially and at weaning) and ADG (Table 4). Furthermore, sows fed with 0.5% BSFLM showed a tendency (*p* > 0.1) to have increased protein and fat % in milk at week 1 (Table 5), whereas the serum concentration of IGF-1 was significantly *(p* < 0.05) increased in the BSFLM1 group compared to the BSFLM2 group initially; it was equalized at weaning (Table 6).

## 4. Discussion

This study evaluated the effects of partial replacement of SBM with BSFLM in the diets of sows. BSFLM was included at 0.5% and 1% of the total diet, and its effects were assessed on sow and their offspring performance. Previously, Kawasaki et al. [15] investigated the effects of substituting conventional protein resources in sow and their piglets and found no notable disparities in sow, while the BW gain of piglets at weaning was comparatively lower in piglets born to sows fed with BSF. Considering these findings, the lack of significant effects observed in our study may be attributed to the late introduction of the experimental diets, which likely limited their influence on reproductive outcomes. Accordingly, dietary treatment did not significantly affect litter size, the number of live or stillborn piglets, or pre-weaning mortality. Our results are consistent with earlier reports [17,18], indicating that short-term dietary interventions during late gestation exert minimal effects on reproductive performance in sows. Given that BSFLM is a rich source of protein and energy [19], its inclusion was expected to maintain a performance comparable to that of SBM without adverse effects, which was confirmed in this study. Although some differences were observed in sow BW both before farrowing and at weaning, these variations appeared to originate from the initial differences at the start of the trial. Therefore, inclusion of BSFLM up to 1% of the total diet did not alter sow BW during gestation or lactation. Similarly, ADFI was not affected, suggesting that BSFLM did not compromise feed palatability or nutrient utilization efficiency. BFT is a crucial indicator of sow body condition, influencing farrowing ease, subsequent estrus, and lifetime productivity [20]. Optimal BFT at farrowing ranges between 14 and 18 mm supports reproductive efficiency [21]. Excessive fatness can predispose sows to dystocia, metabolic disorders, and irregular estrous cycles [22], whereas insufficient BFT (<12 mm) has been associated with extended weaning-to-estrus intervals and reduced reproductive longevity [23]. In the present study, the initial BFT values were within the optimal range for all treatments. Interestingly, sows fed with 1% BSFLM exhibited slightly higher BFT (15.2 mm) than those fed 0.5% BSFLM (14.3 mm) and CON (14.8 mm). This increase could be attributed to the higher lipid content of BSFLM, particularly its rich medium-chain fatty acid (lauric acid) profile [16], which may enhance lipid deposition during lactation. Previous studies have also reported increased intramuscular fat and activation of fat-synthesis-related genes in pigs fed with full-fat BSFLM diets [24,25]. The tendency to improve milk fat and protein content in BSFLM1 sows might explain the slightly lower BFT in this group, as greater nutrient mobilization for milk synthesis can deplete body reserves.

Piglet performance reflected the nutritional influence of BSFLM-fed sows. Piglets from the BSFLM1 group showed higher birth weights compared with those from the control group, although the mechanism remains unclear due to the short feeding duration. A longer feeding period during gestation may be necessary to verify whether BSFLM directly influences fetal growth. Interestingly, weaning BW and ADG of piglets tended to increase in the BSFLM1 group. This improvement may be associated with enhanced milk composition, particularly in the elevated fat and protein levels, which supply essential energy and amino acids for early growth [26]. We hypothesized that the presence of medium-chain fatty acids, such as lauric acids, in BSFLM may facilitate rapid digestion and oxidation, thereby improving energy availability to the offspring [27,28]. However, future studies including digestibility and milk fatty acid analyses are needed to elucidate the exact mechanism of action. Blood biochemical analysis revealed no significant changes in serum calcium, phosphorus, immunoglobulin G (IgG), or cortisol concentrations among treatments, suggesting that BSFLM did not induce metabolic stress in sows. Initially, serum IGF-1 levels were lower in the BSFLM2 sows but normalized by weaning. IGF-1 is a key regulator of embryonic development and lactation, and reductions in its concentration may reflect catabolic states or undernutrition [29,30,31]. The recovery of IGF-1 in the BSFLM2 group indicates that the inclusion of BSFLM may help maintain metabolic and hormonal balance during the demanding lactation phase, likely due to its balanced nutrient profile.

## 5. Conclusions

Our study shows that partially replacing soybean meal with 0.5–1% BSFLM in sow diets does not negatively affect reproductive performance. Instead, BSFLM supplementation tended to improve milk composition and support better piglet growth. Therefore, including BSFLM at these low levels appears to be a safe and nutritionally beneficial alternative to soybean meals.

## Figures and Tables

**Table 1:** Major fatty acid, amino acid, and mineral components of black soldier larva fly meal (BSFLM).

Fatty Acids (g/100 g Fat)	Composition
Lauric acids (C12:0)	42.5
Myristic acids (C14:0)	6.9
Palmitic acids (C16:0)	11.2
Stearic acids (C18:0)	1.4
Oleic acids (C18:1 u9)	12.3
Linoleic acids (C18:2 u6)	3.6
Linolenic acids (C18:3 u6)	0.8
Eicosapentaenoic acids (C20:5 u3)	1.5
Docosahexaenoic acids (C22:6)	0.6
Amino acids (g/100 g protein)	
Histidine	4.10
Threonine	4.49
Arginine	5.22
Tyrosine	7.66
Methionine	2.74
Valine	5.80
Phenylalanine	6.24
Isoleucine	5.08
Leucine	8.81
Lysine	6.44
Serine	4.08
Glysine	4.49
Alanine	5.85

**Table 2:** Composition of sow diets (as-fed basis).

Items	Dietary Treatment		
	CON ^1^	BSFLM1 ^1^	BSFLM2 ^1^
Ingredient, %			
Corn	43.96	44.13	44.28
Wheat	17.00	17.00	17.00
Wheat bran	10.00	10.00	10.00
Palm kernel meal	2.00	2.00	2.00
Soybean meal	7.54	6.85	6.20
Dehulled soybean meal	10.00	10.00	10.00
Molasses	2.00	2.00	2.00
Tallow	3.69	3.66	3.63
Monocalcium phosphate	1.15	1.15	1.15
Limestone	1.20	1.20	1.20
MgO	0.02	0.02	0.02
Salt	0.50	0.50	0.50
Methionine (99%)	0.01	0.02	0.02
Threonine (99%)	0.14	0.15	0.16
L-Lysine (78%)	0.27	0.30	0.32
Vit / Mineral premix ^2^	0.40	0.40	0.40
Choline (25%)	0.12	0.12	0.12
Black soldier fly	-	0.50	1.00
Total	100.00	100.00	100.00
Analyzed value			
Crude protein, %	16.33	16.20	16.28
ME, kcal/kg	3282	3280	3299
Calcium, %	0.77	0.78	0.80
Phosphorus, %	0.74	0.70	0.71
Lys, %	0.96	0.99	0.96
Met, %	0.27	0.26	0.26
Thr, %	0.62	0.64	0.65

^1^ Abbreviations: CON, basal diet; BSFLM1, basal diet soybean meal replaced by 0.5% black soldier fly larva meal (BSFLM); BSFLM2, basal diet soybean meal replaced by 1% BSFLM. ^2^ Provided per kg of complete diet: 16,800 IU vitamin A; 2400 IU vitamin D_3_; 108 mg vitamin E; 7.2 mg vitamin K; 18 mg Riboflavin; 80.4 mg Niacin; 2.64 mg Thiamine; 45.6 mg D-Pantothenic; 0.06 mg. Cobalamine; 12 mg Cu (as CuSO_4_); 60 mg Zn (as ZnSO_4_); 24 mg Mn (as MnSO_4_); 0.6 mg I (as Ca(IO_3_)_2_); 0.36 mg Se (as Na_2_SeO_3_).

**Table 3:** Effect of dietary BSFLM on reproduction performance in sows ^1^.

Items	CON	BSFLM1	BSFLM2	SEM ^2^	*p*-Value
Parity	3.3	3.2	3.2	-	-
Litter size					
Total birth, head	11.8	11.2	11.8	0.9	0.615
Mummification, head	0.0	0.2	0.2	0.1	0.248
Stillbirth, head	0.7	0.3	0.2	0.3	0.228
Total alive, head	11.2	10.7	11.5	0.9	0.545
SUR1 ^3^, %	94.32	96.26	97.05	2.04	0.365
Underweight piglet	1.67	1.67	1.50	0.41	0.428
Body weight, kg					
Initial	200.3 ^ab^	194.0 ^b^	201.7 ^a^	2.4	0.043
Weaning	168.1 ^ab^	160.2 ^b^	170.8 ^a^	2.7	0.019
Body weight difference 1 ^4^	32.2	33.8	30.9	1.2	0.107
Backfat thickness, mm					
Initial	19.3	19.2	19.5	0.3	0.433
After farrowing	17.3	17.0	17.5	0.2	0.158
Weaning	14.8 ^ab^	14.3 ^b^	15.2 ^a^	0.3	0.043
Backfat thickness difference 1 ^5^	2.0	2.2	2.0	0.3	0.677
Backfat thickness difference 2 ^5^	2.5	2.7	2.3	0.3	0.433
ADFI, kg					
Pregnant	2.92	2.83	2.89	0.05	0.204
Lactation	7.29	7.20	7.33	0.04	0.197
Estrus interval	4.2	4.0	4.5	0.5	0.516

^1^ Abbreviations: CON, basal diet; BSFLM1, basal diet soybean meal replaced by 0.5% black soldier fly larva meal (BSFLM); BSFLM2, basal diet soybean meal replaced by 1% BSFLM. ^2^ Standard error of means. ^3^ SUR1: Survival rate of number of alive pigs per number of total born pigs. ^4^ Body weight difference: 1, initial to weaning. ^5^ Backfat thickness difference: 1, initial to after farrowing; 2, after farrowing to weaning. ^a,b^ Means in the same row with different superscript differ significantly (*p* < 0.05).

**Table 4:** Effect of dietary BSFLM on growth performance of suckling piglets ^1^.

Items	CON	BSFLM1	BSFLM2	SEM ^2^	*p*-Value
SUR2 ^3^, %	95.58	96.97	98.61	1.98	0.304
Body weight, kg					
Birth weight	1.19 ^b^	1.34 ^a^	1.26 ^ab^	0.05	0.046
Weaning	6.21	6.49	6.13	0.11	0.098
Average daily gain, g	239	245	232	5	0.073

^1^ Abbreviations: CON, basal diet; BSFLM1, basal diet soybean meal replaced by 0.5% black soldier fly larva meal (BSFLM); BSFLM2, basal diet soybean meal replaced by 1% BSFLM. ^2^ Standard error of means. ^3^ SUR2: survival rate during lactation. ^a,b^ Means in the same row with different superscript differ significantly (*p* < 0.05).

**Table 5:** Effect of dietary BSFLM on milk profile in sow ^1^.

Items	CON	BSFLM1	BSFLM2	SEM ^2^	*p*-Value
Colostrum					
Fat, %	3.86	3.80	3.92	0.15	0.771
Protein, %	12.99	13.27	13.75	0.27	0.108
Lactose, %	2.84	2.78	2.89	0.29	0.793
Solids not fat, %	19.81	19.45	20.18	0.41	0.254
Total-solids, %	23.21	22.87	23.04	0.12	0.109
Frozen point, °C	−0.55	−0.55	−0.55	-	-
Week 1					
Fat, %	9.50	10.49	10.03	0.35	0.093
Protein, %	4.33	4.72	4.48	0.14	0.099
Lactose, %	5.11	4.86	4.98	0.22	0.445
Solids not fat, %	10.27	10.42	10.12	0.50	0.685
Total-solids, %	19.57	20.01	19.80	0.47	0.543
Frozen point, ℃	−0.62	−0.62	−0.62	-	-

^1^ Abbreviations: CON, basal diet; BSFLM1, basal diet soybean meal replaced by 0.5% black soldier fly larva meal (BSFLM); BSFLM2, basal diet soybean meal replaced by 1% BSFLM. ^2^ Standard error of means.

**Table 6:** Effect of dietary BSFLM on blood profile in sow ^1^.

Items	CON	BSFLM1	BSFLM2	SEM ^2^	*p*-Value
Initial					
Calcium, mg/dL	9.38	9.93	9.33	0.18	0.096
Phosphorus, ng/mL	6.80	7.48	6.90	0.25	0.104
IgG, mg/dL	297.0	268.8	247.3	28.9	0.264
IGF-1, ng/mL	64.28 ^ab^	81.63 ^a^	60.50 ^b^	5.38	0.032
Cortisol, µg/dL	2.94	3.20	1.71	0.84	0.258
Weaning					
Calcium, mg/dL	9.45	9.58	9.35	0.31	0.622
Phosphorus, ng/mL	6.63	6.68	6.33	0.71	0.774
IgG, mg/dL	470.5	445.3	554.5	89.7	0.151
IGF-1, ng/mL	83.48	85.20	93.80	11.98	0.564
Cortisol, µg/dL	3.49	3.06	2.09	0.98	0.351

^1^ Abbreviations: CON, basal diet; BSFLM1, basal diet soybean meal replaced by 0.5% black soldier fly larva meal (BSFLM); BSFLM2, basal diet soybean meal replaced by 1% BSFLM. ^2^ Standard error of means. ^a,b^ Means in the same row with different superscript differ significantly (*p* < 0.05).

## Data Availability

The data presented in this study are available on request from the corresponding author and the data is not publicly available due to privacy and ethical restrictions.
